# Belief Interval of Dempster-Shafer Theory for Line-of-Sight Identification in Indoor Positioning Applications

**DOI:** 10.3390/s17061242

**Published:** 2017-05-30

**Authors:** Jinwu Wu, Tingyu Zhao, Shang Li, Chung-Ming Own

**Affiliations:** School of Computer Software, Tianjin University, Tianjin 300350, China; wu_jinwu@outlook.com (J.W.); tingyuzhao@tju.edu.cn (T.Z.); lishang0036@tju.edu.cn (S.L.)

**Keywords:** location estimation, NLOS, dempster-shafer theory, belief interval

## Abstract

Location data are among the most widely used contextual data in context-aware and ubiquitous computing applications. Numerous systems with distinct deployment costs and levels of positioning accuracy have been developed over the past decade for indoor positioning purposes. The most useful method focuses on the received signal strength (RSS) and provides a set of signal transmission access points. Furthermore, most positioning systems are based on non-line-of-sight (NLOS) rather than line-of-sight (LOS) conditions, and this cause ranging errors for location predictions. Hence, manually compiling a fingerprint database measuring RSS involves high costs and is thus impractical in online prediction environments. In our proposed method, a comparison method is derived on the basis of belief intervals, as proposed in Dempster-Shafer theory, and the signal features are characterized on the LOS and NLOS conditions for different field experiments. The system performance levels were examined with different features and under different environments through robust testing and by using several widely used machine learning methods. The results showed that the proposed method can not only retain positioning accuracy but also save computation time in location predictions.

## 1. Introduction

Location awareness has become an essential aspect of wireless communication systems and has enabled a myriad of commercial and military applications [1]. Positioning and navigation services have gradually become involved in various activities of daily living for many people worldwide. This usage is expected continue in the future, with more people becoming direct and indirect users of such services. Because of the high demand for location awareness, high-availability wireless broadband communications featuring precise position prediction are becoming extremely prevalent.

Recently, the problem of position estimation has been thoroughly studied in line-of-sight (LOS) environments, which involve Gaussian measurement errors. In various crowded situations, such as indoor environments and some urban areas, the measurement errors are non-Gaussian and heavy-tailed; consequently, estimation performance is seriously affected by methods premised on Gaussian assumptions [2]. However, in practical situations, signal acquisition [3], multiple-user interference [4], multipath effects [5], and non-line-of-sight (NLOS) [6] are the challenges remaining in traditional localization and communication applications. In particular, the NLOS issue is critical for high-resolution localization systems, because NLOS propagation involves positive biases in distance evaluation algorithms. Generally, NLOS conditions frequently occur in enclosed areas, crowded environments, and urban canyons.

To reduce the negative influence of multipath effects, the implemented system must identify and mitigate NLOS conditions. Usage of LOS/NLOS identification methods can greatly improve a system’s ability to locate people and objects inside buildings. Generally, the received signal strength (RSS) in LOS situations is considerably stronger than that in NLOS situations, which can cause large distance estimation errors when implementing propagation systems. In practice, even when NLOS conditions are identified, multipath effects complicate acquisition of accurate path loss models. Therefore, other methods beyond simple propagation models, such as Gaussian regression, should be employed to estimate distances [7].

Although many theoretical models have been studied for LOS/NLOS propagation, a practical LOS identification scheme requires channel profiles, which involves dedicated channel sounders or assumes abundant random noise that can effectively be processed with statistical models [8]. Regarding more pervasive solutions, most existing approaches either employ extremely wideband signals, such as ultrawide band (UWB) [9], or resort to relatively long-range communications such as in cellular networks [10]. Because of the large bandwidth of UWB signals, the LOS component can be readily identified and extracted from the received signal. Furthermore, the commonly employed techniques in NLOS identification of UWB signals are hypothesis testing and the machine learning of features from received signals [11,12]. Features such as range estimates, channel statistics, and position estimation differ greatly between LOS and NLOS components. However, considering an indoor positioning service with meter-level accuracy and resolution paths with a bandwidth of ≤15 m, typical mobile wireless operations within the bandwidth of 20 MHz cannot resolve bandwidth mismatches, which hampers direct adoption of geographic space methods [13].

In the present study, to improve the identification of NLOS situations, a novel approach to DS theory’s rule of combination is proposed to support the classification of key features from various collected RSS measurements useful for identifying NLOS conditions, including the mean, standard deviation, kurtosis, skewness, and Rician K factor. Each feature is derived from a set of series of RSS samples collected from the access point (AP; or beacon) at a particular location over a short period. Furthermore, a new Dempster-Shafer (DS) detection approach is presented—specifically, a noise detection algorithm for building decision rules. To obtain a decision rule, bodies of evidence are extracted and their mass functions are defined using local information in the time series data. The proposed method is based on a fingerprinting localization system that has had limited performance in the past; with a new feature extraction framework, the proposed method can identify the original signals in the obstacle environment and improve the localization precision stability. Although our proposed method has suffered the difficulties on the construction of the offline database, just like the traditional fingerprinting method, however, with the flexibility of belief interval, and the increasing with the feature dimensions, our system performance achieves the improvement on the indoor localization.

The remainder of this paper is organized as follows: in Section 2, some basic concepts of RSS signals are reviewed, and DS theory is also introduced. Section 3 describes the design of a new type of belief interval in detail. In Section 4, data from extensive field experiments are presented to demonstrate that the proposed method outperforms other decision-based methods. Finally, the conclusion is given in Section 5.

## 2. Proposed Framework

### 2.1. The Features of RSS

Numerous studies examining indoor positioning systems have determined Bluetooth RSS properties. This type of research has shown that user orientation can cause a variation of up to 5 dBm in the received signal strength indicator (RSSI) level [14]. At any location, the various orientations of users and mobile devices relative to the transmitter could cause the mean RSS value to change. The modeling of RSS-based location fingerprinting is essential for location determination algorithms; examples of RSS-based location fingerprinting models include the probabilistic approach model and preliminary analytical model [2]. Previously, Gaussian or lognormal distributions have been used to model the randomness of RSS. For example, in [14], a large-scale measurement was provided to summarize evidence that most RSS histograms could be fitted appropriately with Gaussian distributions whereas few histograms could be fitted with bimodal Gaussian distributions. Other revealing examples are discussed in [15,16,17], where the Wang research team had done the subarea division for the RSS fingerprint, and applied this idea to solve the problems of crowdsourcing, clustering and matching on the RSS measurements and position prediction. Their proposed localization scheme increased the localization accuracy compared with the classical fingerprinting method.

**Figure 1:**
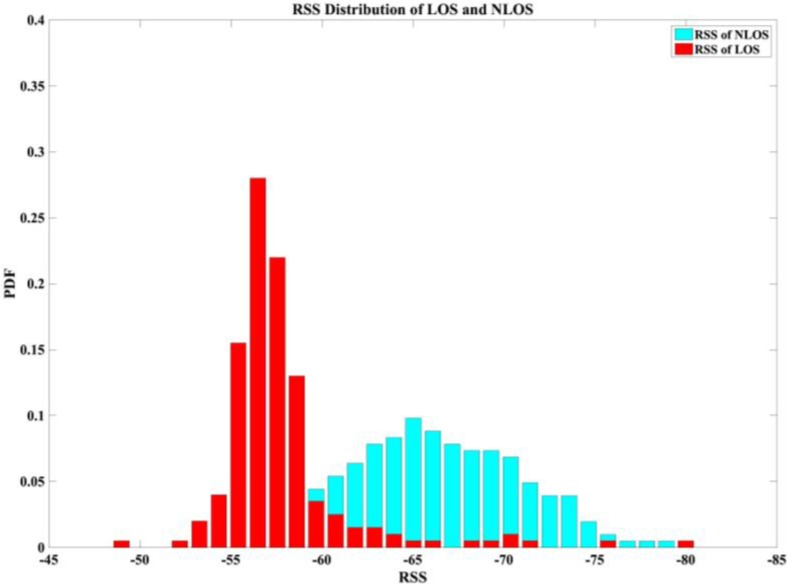
The period sampling of RSS value in LOS/NLOS conditions.

Current signal-based RSS location systems have two problems. First, a considerable manual calibration effort is required to construct a radio map in the offline training phase; second, the positioning accuracy varies with the environmental dynamics. Three dynamic factors that change frequently over time in the environment were proposed in [18], namely the presence of people, relative humidity, and movement. These factors easily affect the radio signals propagating from APs to mobile devices and are responsible for changes in positioning accuracy. The RSS values calibrated previously in the radio map may be outdated; this condition degrades the positioning accuracy because of the presence of people. Furthermore, some studies have minimized the distance estimation residual by selecting the optimal subset from available APs, and they have also improved the algorithm to reduce the computational complexity by using the three distance measurements instead of using derived distances [19,20,21]. However, these techniques are all problematic for considerations of LOS and NLOS APs; combined-signal situations cause additional problems. For example, a generic NLOS mitigation technique was applied to wireless systems to reduce the NLOS distance estimation error by a convex algorithm [22]. That approach was unrealistic because it required 50% more LOS samples compared with other approaches. Figure 1 depicts the period sampling of RSS values in the fixed point; the data show the apparent instability in LOS/NLOS conditions.

**Figure 2:**
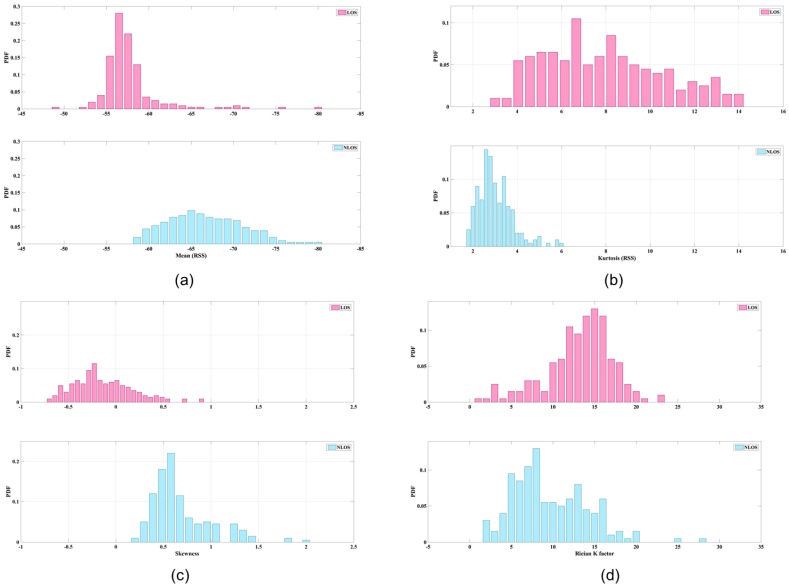
The feature demonstrations of the LOS/NLOS signal, (**a**) mean value; (**b**) Kurtosis value; (**c**) Skewness value; (**d**) Rician K factor.

Through observation of multiple RSS signals in LOS and NLOS conditions, we observed several key features from the RSS measurements useful for identifying LOS and NLOS conditions, including the mean, standard deviation, kurtosis, skewness, Rician K factor, and log mean. Each feature was obtained from a set of time series *t* signals [$p^{1},p^{2},\ldots p^{\left(t\right)}]$ in a fixed location over a short period (e.g., 4 s) from one AP. All LOS/NLOS identification and mitigation algorithms in our study are based on this assumption, and the features are introduced as follows:

- Mean and standard deviation ($\mu ,\;\sigma^{2})$ are derived from RSS data. The average strength levels of these two types of data can be categorized for some situations. Figure 2a illustrates the mean values of LOS and NLOS conditions; the clarity of the data is striking. The maximum LOS value is greater than the maximum NLOS value.
- Kurtosis (κ) is used to measure the sharpness of the peak of the probability distribution. The measurements of RSS in LOS conditions are more centralized than those derived from NLOS conditions, because the dominant LOS signal has a stronger energy presentation. Figure 2b shows an example of the kurtosis data. Because of the representation of numbers at the peaks of probability distributions, the figure is based on the number of samples. If fewer samples had been collected, it would be difficult to ascertain the different conditions.
- Skewness (*S*) is used to measure the asymmetry of the probability distribution. Because of multipath considerations, LOS signals tend to decay following a Rayleigh distribution, whereas NLOS signals tend to follow a Rician distribution. Accordingly, the skewness of a Rayleigh distribution is a generally larger than that of a Rician distribution; hence, a typical LOS measurement should have lower skewness than a typical NLOS measurement. Figure 2c shows an example of skewness data.
- Rician K factor ($K_{r})$ is defined as the ratio of power of the direct path to the power of other scattered paths. An empirical study demonstrated a positive relationship between the Rician K factor and an LOS signal [23]. The probability density function of the Rician K factor is defined as follows:
  (1) $$p\left(r\right)=\frac{2{\left(K_{r}+1\right)}^{r}}{\Omega }\exp\left(-K_{r}-\frac{{\left(K_{r}+1\right)}^{2}}{\Omega }\right)\cdot I_{0}\left(2r\sqrt{\frac{K_{r}\left(K_{r}+1\right)}{\Omega }}\right)$$
  where $I_{0}$ is a first class zeroth-order modified Bessel function, r is signal envelope, and Ω is defined as equal to $A^{2}+\sigma^{2}$. According to our test, the results of which are shown in Figure 2d, the centralized Rician K factor of an LOS value is larger than that of an NLOS value.
- Log mean (ℒ) is used primarily for NLOS mitigation. According to our observations, the relationship of RSS and log mean can be illustrated clearly.

### 2.2. Basic Principles of Dempster-Shafer Theory

The Dempster-Shafer theory (D-S theory) is capable of deriving probabilities for a collection of hypotheses, on the contrary, the classical probability theory is fixed on a single hypothesis. Because the D-S theory allows the system inferencing with the imprecision and uncertainty, it is considered to be a more flexible than the traditional probability methods. Particularity, the most remarkable advantage of the D-S theory is to deal with the missing information. That is, this theory is capable to deal uncertain condition in our signal environment.

Suppose Θ is the hypotheses space named the frame of discernment. These singleton hypotheses are assumed to be mutually exclusive. The D-S theory can consider the subset of Θ. Then a mass function *m* can be depicted on the power set of Θ, $2^{\Theta }$ as

$$m:2^{\Theta }\to \left[0,\;1\right].$$

The *m* function is for every element *A* of $2^{\Theta }$, hence, the mass value *m*(*A*) belongs to the [0–1] interval as,

m(∅)=0,

$$\sum_{A\in 2^{\Theta }}m\left(A\right)=1,\;$$

where ∅ is an empty set [24]. The focal element of *m* is used to represent a measure of belief exactly to *A*, it denoted as m(A)>0. Besides, *m*(Θ) = 1 and *m*(A:A≠Θ) = 0 are represented as the global ignorance by the weight of evidence is not identified among the hypotheses.

Accordingly, to represent the imprecision and uncertainty by the mass function, in [25], two functions belief (*Bel*) and plausibility (*Pls*) are both derived as follows,

$$\{\begin{array}{c} Bel\left(\emptyset \right)=0,\\ Bel\left(A\right)=\sum_{B\subseteq A}m\left(B\right), \end{array}\{\begin{array}{c} Pl\left(\emptyset \right)=0,\\ Pl\left(A\right)=\sum_{B\cap^{\text{​}}A\neq \emptyset }m\left(B\right), \end{array}$$

In our study, we referred to the lower and upper bound on the probability as the belief and plausibility functions. Thus, “belief interval” is often called by the [*Bel*(*A*), *Pl*(*A*)], and the length of interval can be interpreted as the degree of uncertainty of *A*.

### 2.3. Belief Interval Comparison

After applying Dempster’s rule to each hypothesis of different bodies of evidence, the new mess is computed by the belief and plausibility values. However, the main issue is on the criterion by the hypothesis we will made. That is, it is involved in developing a “decision rule” for the final decision. Many applications prefer a singleton hypothesis as their final decision. There are three widely used decision rules: (a) maximum belief; (b) maximum plausibility; and (c) maximum belief without overlapping of belief interval [25]. In our study, interval comparison is an essential idea for the system developing. Theoretically, intervals can only be partially ordered and hence cannot be compared in ordinary sense. However, if intervals are used in applications, the flexibility in the interval will be retained, and the comparison of them becomes necessary.

**Figure 3:**
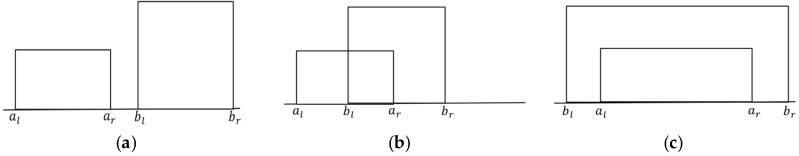
The example of interval relations, (**a**) is the exclusion case; (**b**) is the overlapping case and (**c**) is the inclusion case.

The example of interval relations is shown in Figure 3, including exclusion, overlapping and inclusion cases. Let *A* = [$a_{l},\;a_{r}]$ and *B* = [$b_{l},\;b_{r}]$ be independent intervals and $a\in \left[a_{l},\;a_{r}\right]$, $b\in \left[b_{l},\;b_{r}\right]$ be random values distributed on these intervals. Accordingly, Dymova et al. defined four mutually exclusive events $H_{i}$, *i =* 1 to 4, may take place in considered situation in Figure 3 [26]. In the exclusion case, the event is depicted as follows:

$$H_{1}:a\in \left[a_{l},\;a_{r}\right]\&\;b\in \left[b_{l},b_{r}\right],$$

in the Dempster notation, we obtained

(2) $$m\left(\left\{A<B\right\}\right)=P\left(H_{1}\right)=1$$

For the overlapping case, four events are described as:

$$H_{1}:a\in \left[a_{l},\;b_{l}\right]\&\;b\in \left[a_{r},b_{r}\right],\;H_{2}:a\in \left[a_{l},\;b_{l}\right]\&\;b\in \left[b_{l},a_{r}\right],\;$$

$$H_{3}:a\in \left[b_{l},\;a_{r}\right]\&\;b\in \left[b_{l},b_{r}\right],\;H_{4}:a\in \left[b_{l},\;a_{r}\right]\&\;b\in \left[a_{r},b_{r}\right],$$

in the Dempster notation, we obtained

(3) $$m\left(\left\{A<B\right\}\right)=P\left(H_{1}\right)+P\left(H_{2}\right)+P\left(H_{4}\right),$$

(4) $$m\left(\left\{A<B,A=B\right\}\right)=P\left(H_{3}\right).$$

Finally, for the inclusion case, three events are listed as follows,

$$H_{1}:a\in \left[a_{l},\;a_{r}\right]\&\;b\in \left[b_{l},a_{l}\right],\;H_{2}:a\in \left[a_{l},\;a_{r}\right]\&\;b\in \left[a_{l},a_{r}\right],$$

$$H_{3}:a\in \left[a_{l},\;b_{l}\right]\&\;b\in \left[a_{r},b_{r}\right],\;$$

in the Dempster notation, the evidence value is obtained as

(5) $$m\left(\left\{A>B\right\}\right)=P\left(H_{1}\right),$$

(6) $$m\left(\left\{A=B\right\}\right)=P\left(H_{2}\right),$$

(7) $$m\left(\left\{A<B\right\}\right)=P\left(H_{3}\right).$$

## 3. System Design

**Figure 4:**
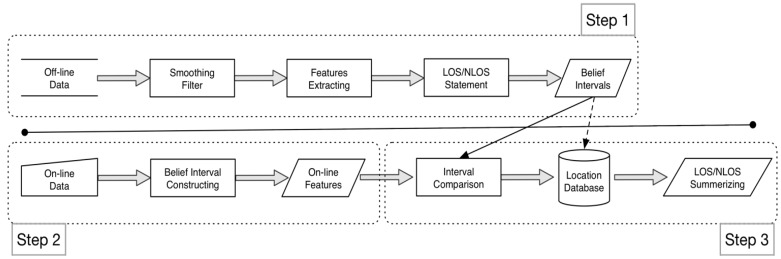
The proposed system structure.

Figure 4 depicts the proposed system structure. First, in the offline phase, the system gathers advanced RSS offline data, and the features of LOS and NLOS data are extracted during this phase. Accordingly, in the online phase, when a user stands in a fixed position, the system collects the online RSS value and extracts the online features. Finally, the proposed mass function is applied and compared for the LOS and NLOS conditions.

The steps performed for our proposed system are listed as follows:

Step 1. In the office system phase, like the fingerprint method, we record four directions of RSS signals in a time period at each location, the direction back to the beacon is the collection of NLS, the other three directions are arranged to the collection of LOS.
  - Compute the basic assignments of feature *f* for each location *p*, that is $m_{f,L}\left(p\right)$, $m_{f,NL}\left(p\right)$ and $m_{f,L\&N}\left(p\right)$. Accordingly, transfer the assignments to the Belief Interval of LOS and NLOS, that are $B_{f,L}\left(p\right)$ and $B_{f,NL}\left(p\right)$ respectively.
Step 2. In the online system phase, the user’s position will be predicted by holding the phone, and receive a series of RSS values. Then, derive the online Belief Interval of LOS and NLOS, that is $B_{f}\left(t\right)$, *t* is the time period *t*.
Step 3. In the belief interval comparison phase, the system compared the online interval to the offline intervals by the proposed method of $CF_{A\bar{A}}$, where $A\;\mathrm{and}\;\bar{A}$ are two comparing intervals. The prediction location p is obtained by the following equation:
  $$p=\arg_{i}\mathrm{Max}\;\sum_{i\in S}CF_{B_{f}\left(t\right),\;B_{f,L}\left(i\right)}+CF_{B_{f}\left(t\right),\;B_{f,NL}\left(i\right)}\;,$$
  where *S* is set of locations in the field experiment.

### 3.1. The Offline System Phase

During this phase, the system collects and extracts the RSS features for further processing. According to the RSS symbolization, the proposed system adopts two filters to smooth the LOS and NLOS data. All of the data are collected in a fixed position; the difference of LOS and NLOS is calculated on the disturbances of the human body. To validate NLOS signal blocking, the signal-receiving device is tested with full obstruction by a standing human body, whereas to test LOS transmission, the user must hold the device against the flat side of the front of the body, near the chest.

**Figure 5:**
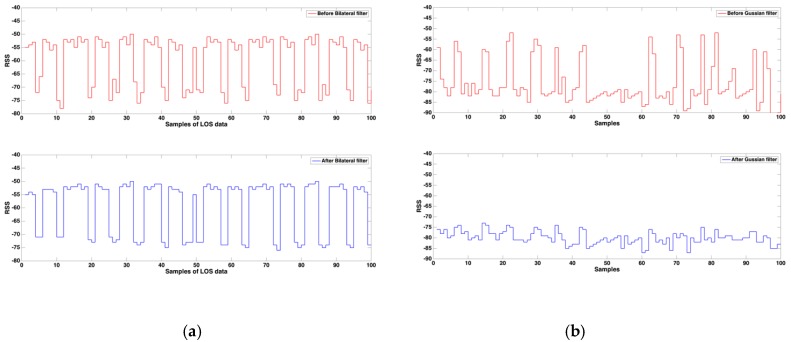
The filtering demonstration of (**a**) Bilateral filter for the LOS, and (**b**) Gaussian filter for the NLOS data.

Regarding LOS signatures, the performance of data transmission is straightforward; data cluster together. Most RSS histograms could be fitted very accurately with a Gaussian distribution. Thus, in a series of time domain RSS data, the maximum and minimum strength values of power can be ignored. In our study, the proposed system applies a bilateral filter on the RSS in the LOS condition. The domain filter is a traditional filtering method; it enforces closeness by weighing pixel values with coefficients that become smaller with longer distances. Similarly, the range filter averages image values with weights that decay with dissimilarity. Hence, to combine the domain and range filters, the bilateral filter can detect the geometric looseness and photometric similarity, and can prefer near values to distant values in both domain and range [27]. The equation of the bilateral filter is listed as follows; p is represented as the predicated location surrounded by the |*S*| signals:

(8) $$m_{f,L}\left(p\right)=\frac{1}{\left|S\right|}\sum_{i\in S,\;i\neq p}\exp\left(-\frac{{\left(x_{p}-x_{i}\right)}^{2}+{\left(y_{p}-y_{i}\right)}^{2}}{2\sigma_{s}^{2}}-\frac{{\left|r_{p}-r_{i}\right|}^{2}}{2\sigma_{r}^{2}}\right),$$

which is used to represented as the basic LOS feature assignment in position p. ($x_{p},\;y_{p}$) are used to represent as the x and y coordinates of the location, besides, $r_{p}\;$is used to indicate the RSS value in location p, $\sigma_{S}$ and $\sigma_{r}$ are derived as the standard deviation of the surrounded signals with distance and RSS value respectively. In (8), the most similar RSS value inside the main area of p must be filtered first. Thus, the LOS data will gather together. Figure 5a demonstrates the filtering performance. However, the RSS value is more unstable in the NLOS condition than in the LOS condition, and the NLOS value is much smaller than the LOS value. In our proposed system, the Gaussian filter is applied to the NLOS condition data. According to Figure 5b, the sampling feature of the NLOS data is in the peak shape calibration. The Gaussian filter is a linear smoothing filter, which has the properties of having no overshoot to a step function input and minimizing the rise and fall time. Hence, Equation (9) is defined as follows in our system:

(9) $$m_{f,NL}\left(p\right)=\frac{1}{\left|S\right|}\sum_{i\in S,i\neq p}\exp\left(-\frac{{\left|r_{p}-r_{i}\right|}^{2}}{2\sigma_{r}}\right),$$

where $m_{f,NL}\left(p\right)$ is the basic NLOS feature assignment of *f* feature in position *p*. Using these two filters for the RSS data in location p can strength the difference area between NLOS and LOS conditions.

In the general fingerprint database, the basic solution procedure is to compare the RSS values from online to offline; the place with the minimum differential value is the predicted location. Furthermore, the difference between the online and offline RSS values is always valid. In the present study, according to the features statement in Section 2, the features of LOS and NLOS signals were collected and computed in each location p; that is, the assessment matrix of each feature *f* can be listed as follows:

(10) $$M_{f}=\left[\begin{array}{c} \begin{array}{ccc} m_{f,L}\left(1\right) & m_{f,NL}\left(1\right) & m_{f,L\&N}\left(1\right) \end{array}\\ \begin{array}{ccc} m_{f,L}\left(2\right) & m_{f,NL}\left(2\right) & m_{f,L\&N}\left(2\right) \end{array}\\ \begin{array}{ccc} \begin{array}{c} \begin{array}{c} \vdots \\ m_{f,L}\left(p\right)\\ \vdots \end{array}\\ m_{f,L}\left(N\right) \end{array} & \begin{array}{c} \begin{array}{c} \vdots \\ m_{f,NL}\left(p\right)\\ \vdots \end{array}\\ m_{f,NL}\left(N\right) \end{array} & \begin{array}{c} \begin{array}{c} \vdots \\ m_{f,L\&N}\left(p\right)\\ \vdots \end{array}\\ m_{f,L\&N}\left(N\right) \end{array} \end{array} \end{array}\right],$$

where $m_{f,L}\left(p\right)$, $m_{f,NL}\left(p\right)$, and $m_{f,L\&N}\left(p\right)$ represent the basic feature assignments of LOS, NLOS, and the interfering section between LOS and NLOS in the location p, respectively. The function $M_{f}$ is used to represent the features of mean, kurtosis, skewness, Rician K factor, and log mean at each location as $M_{m}$, $M_{k}$, $M_{S}$, $M_{r}$, $M_{ℒ}$ respectively.

Considering that LOS (*L*) and NLOS (*NL*) are mutually exclusive, the combined basic assignments in location p are denoted by $m_{f,L}\left(p\right)$, $m_{f,NL}\left(p\right),$ and $m_{f,L\&N}\left(p\right)$, and:

(11) $$m_{f,L}\left(p\right)+m_{f,NL}\left(p\right)+m_{f,L\&N}\left(p\right)=1.$$

With the $m_{.}\left(p\right)$ as given previously, the evidence measures for the LOS and NLOS signal status can be assumed as:

(12) $$bel_{f,L}\left(p\right)=m_{f,L}\left(p\right),$$

(13) $$pl_{f,L}\left(p\right)=\alpha \cdot m_{f,L}\left(p\right),$$

(14) $$bel_{f,NL}\left(p\right)=m_{f,NL}\left(p\right),$$

(15) $$pl_{f,NL}\left(p\right)=m_{f,NL}\left(p\right)+m_{f,L\&N}\left(p\right)=1-m_{f,L}\left(p\right).$$

According to our field experiment, the value of α is adaptively in the distinct field. Equations (16) and (17) represent as the basic operation of the belief intervals $B_{f,L}\left(p\right)$ and $B_{f,NL}\left(p\right)$, which are the gathering area of the LOS and NLOS signals, that is:

(16) $$B_{f,L}\left(p\right)=\left[bel_{f,L}\left(p\right),\;pl_{f,L}\left(p\right)\right]=\left[m_{f,L}\left(p\right),\alpha \cdot m_{f,L}\left(p\right)\right],$$

(17) $$B_{f,NL}\left(p\right)=\left[bel_{f,NL}\left(p\right),\;pl_{f,NL}\left(p\right)\right]=\left[m_{f,NL}\left(p\right),\;1-m_{f,L}\left(p\right)\right].\;$$

### 3.2. The Online System Phase

In the online prediction phase, data are collected in a location *i*, for a short period of time no longer than 5 s. To eliminate the unstable broadcasting values at channel 37, 38 and 39, a newly simplified weight ratio method is proposed for our system. According to our system, the first five RSS values are summarized in descending order of the series data. Then, the derived weight is defined as follows:

(18) $$RSS_{max}=Max\left(RSS\left(\tilde{t}\right)\right),$$

and:

(19) $$RSS_{t}=Last\left(RSS\left(\tilde{t}\right)\right),$$

where the function $RSS_{max}$ is the maximum value of a series *RSS* values at the time period $\tilde{t}$, and $RSS_{t}$ is the last received signal value at the time period $\tilde{t}$. To compare the RSS value with different features, the online assignment with features can be derived, and the online belief interval will be obtained as follows,

(20) $$B_{f}\left(t\right)=\left[bel_{f}\left(t\right),\;pl_{f}\left(t\right)\right]=\left[\exp\left(-\frac{RSS_{t}^{2}}{2\sigma_{\tilde{t}}}\right),\exp\left(-\frac{RSS_{max}^{2}}{2\sigma_{\tilde{t}}}\right)\right],$$

where *f* = 1, …, 6 for the six features in our field experiments.

### 3.3. The Belief Interval Comparison

Regarding the interval comparison, Kundu [28] defined the preference relationship between two intervals *A* and *B* on the real line by using the following equation:

(21) $$L\left(A,\bar{A}\right)=\max\left\{0,\;P_{A\bar{A}}\left(x<y\right)-P_{A\bar{A}}\left(x>y\right)\right\},$$

where $P_{A\bar{A}}\left(x<y\right)$ denotes the probability with *x* < *y*, given that x∈A and $y\in \bar{A}$ are uniformly distributed and independently in the intervals *A* and $\bar{A}$. Although Kundu’s approach is intuitively appealing, the main disadvantage is that it is inconsistent with the preferences of a rational decision maker.

In the present study, the interval relations of the exclusion case, overlapping case, and inclusion case were derived using the following equations, where the first interval A is represented as $A=[a_{L},\;a_{R}$] = {*a*:$\;a_{L}\leq a\leq a_{R}\}$, and $\bar{A}=[{\bar{a}}_{L},\;{\bar{a}}_{R}$] =$\;\left\{\;\bar{a}:{\bar{a}}_{L}\leq \bar{a}\leq {\bar{a}}_{R}\right\}$. The midpoint and half-width of interval A are expressed as follows:

(22) $$Mi\left(A\right)=\frac{a_{L}+a_{R}}{2},$$

and:

(23) $$Ha\left(A\right)=\frac{a_{R}-a_{L}}{2}.$$

Hence, to compare the two intervals, in our study, the authors derived the comparison functions CF(·) for the further comparison in the mentioned cases in Section 2.3. That is, the comparison function for the inclusion case is

$$CF\left(A<\bar{A}\right)=\frac{Mi\left(\bar{A}\right)-Mi\left(A\right)}{Ha\left(\bar{A}\right)+Ha\left(A\right)},$$

and the comparison function of the overlapping case is defined as:

$$CF\left(A≅\bar{A}\right)=\frac{2Mi\left(A\right)}{Ha\left(\bar{A}\right)+Ha\left(A\right)},$$

where $A\subset \bar{A}$. Note that there is no needed to derive the comparison of exclusion case. For the purpose of simplifying the computation, the author defined the evaluated function for the three mentioned cases as follows:

(24) $$CF_{A\bar{A}}=\frac{\left(a_{R}-{\bar{a}}_{L}\right)/2}{Ha\left(A\right)+Ha\left(\bar{A}\right)},$$

where $CF_{A\bar{A}}$ is the overlapping indicator of the interval relationship. When $CF_{A\bar{A}}$ < 0, two intervals of *A* and $\bar{A}$ are in the exclusion case; then, two belief intervals have no intersection, and no similarities exist between them. However, when $CF_{A\bar{A}}\geq \frac{Ha\left(A\right)}{Ha\left(A\right)+Ha\left(\bar{A}\right)}$, two intervals of *A* and $\bar{A}$ are in the inclusion case, and $CF_{A\bar{A}}<\frac{Ha\left(A\right)}{Ha\left(A\right)+Ha\left(\bar{A}\right)}$, two intervals of *A* and $\bar{A}$ are in the overlapping case. $CF_{A\bar{A}}$ not only can represented as the degree of similarity between two intervals, but also can simply the computation for the judgement of three mentioned interval cases. Accordingly, the definition can be proved as follows.

**Proposition** **1.** *Let* $A=\left[a_{L},\;a_{R}\right]$ *and* $\bar{A}=\left[{\bar{a}}_{L},\;{\bar{a}}_{R}\right]$*; our proposed definition Equation (24) can indicate the three cases of the intervals’ comparison*.

**Proof.**  There are three cases with exclusion, overlapping, and inclusion for the representing relationship of the belief interval. ☐;

Case 1. The exclusion case: According to Figure 3, no intersection exists between two belief intervals; hence, $a_{R}-{\bar{a}}_{L}<0$, then $CF_{A\bar{A}}\;$< 0.
Case 2. The overlapping case: Equation (24) can be derived as follows:
  $$\frac{\left(a_{R}-{\bar{a}}_{L}\right)/2}{Ha\left(A\right)+Ha\left(\bar{A}\right)}\;=\;\frac{\left(a_{R}-{\bar{a}}_{L}\right)/2+\left(a_{L}-{\bar{a}}_{L}\right)/2}{Ha\left(A\right)+Ha\left(\bar{A}\right)}\;=\;\frac{Ha\left(A\right)-\left({\bar{a}}_{L}-a_{L}\right)/2}{Ha\left(A\right)+Ha\left(\bar{A}\right)}\;<\;\frac{Ha\left(A\right)}{\;Ha\left(A\right)+Ha\left(\bar{A}\right)},$$
  where $b_{L}-a_{L}>0$ in this case.
Case 3. The inclusion case: According to Figure 3, $a_{L}-b_{R}\geq 0$ exists in this case; thus, Equation (24) can be derived as follows:
  $$\frac{\left(a_{R}-{\bar{a}}_{L}\right)/2}{Ha\left(A\right)+Ha\left(\bar{A}\right)}\;=\;\frac{\left(a_{R}-a_{L}\right)/2+\left(a_{L}-{\bar{a}}_{L}\right)/2}{Ha\left(A\right)+Ha\left(\bar{A}\right)}\;=\;\frac{Ha\left(A\right)+\left(a_{L}-{\bar{a}}_{L}\right)/2}{Ha\left(A\right)+Ha\left(\bar{A}\right)}\;\geq \;\frac{Ha\left(A\right)}{Ha\left(A\right)+Ha\left(\bar{A}\right)}$$

## 4. Experiments and Discussions

### 4.1. Experiment Environment Initialization

**Figure 6:**
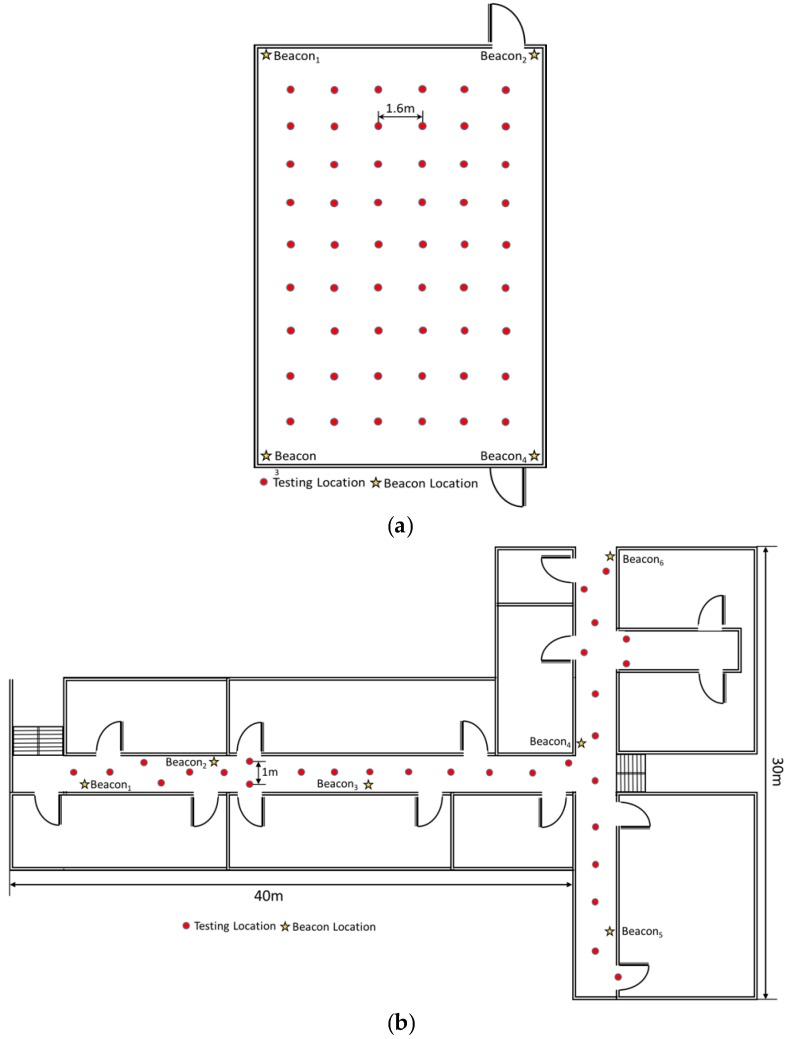
The two environments of field experiment. (**a**) field experiment at 30 m × 30 m square area; (**b**) field experiment at two corridors with 40 and 30 m lengths.

In this section, we report on experiments conducted in two physical environments. One was the area on the third floor of the 55th building of Tianjin University, which had four beacons deployed on the corners of a square area that was 30 m × 30 m (Figure 6a). This area was divided into 54 blocks for signal detection. The testing locations are marked by red dots in Figure 6. The other field experiment was performed in the same building but on the second floor. The second location had two corridors that were 40 and 30 m in length (Figure 6b). The signal detecting points were equally distributed at 1-m intervals along the corridors. The area was a typical building hall with some pillars at each corner of the area. The center of the area was relatively open and people were able to walk around freely. Six Bluetooth transmitters were placed in the corners of the area to ensure full signal coverage of the testing area. Each Bluetooth transmitter was designed to transmit 100 signals per second, and the handheld system used in this study collected 800–1000 RSS samples in each location.

In the offline system phase, because the consideration of interference by human bodies, the data were collected during two periods; the first was 10:00 a.m. (with crowds) and the second was 11:00 p.m. (without crowds).

In the experiments, the accuracy of NLOS identification techniques was subject to interference from people walking around and other signal noise. Although people standing around do not necessarily block LOS signals, they may absorb other components of the received BLE signal, which leads to variation in the measurement distribution. However, from the long-term perspective of practical use, it is impossible to avoid interference by people. Thus, our system supplies two categories of RSS samples in the data sets to account for this type of interference. The first part was collected during nights at approximately 10:00 p.m. when few people were walking around to absorb and block the signals. The other category of samples was collected during the day at approximately 11:00 a.m. when numerous students were walking around the corridors, which interfered with the RSS measures. In our data, each of the two categories contains approximately 1500 sample sets, each of which is composed of 1000 RSS samples. Our system divided sample sets into subsets according to quantities of data, and extracted the features from each subset for the computational analysis. After the offline database of measurements had been built, the test of the accuracy of the proposed method was executed.

### 4.2. The Comparisons on the Distinct Features

**Figure 7:**
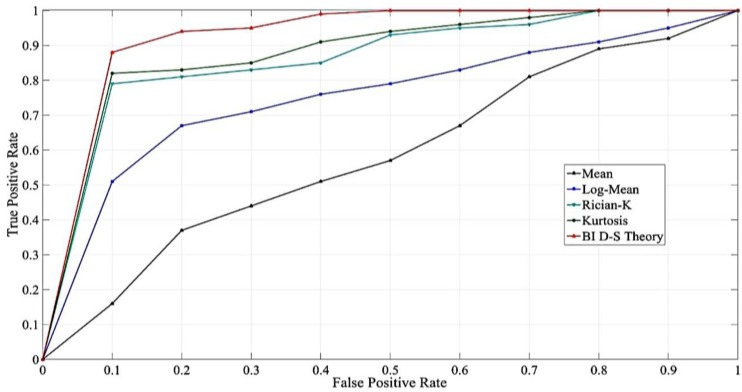
The distinct features comparisons.

To strengthen the features of our proposed method, the performance of the various features was examined, a comparison of which is shown in Figure 7. In this field experiment, we collected 5 s of online data for each testing point, and computed four features of the online data, namely the mean, kurtosis, Rician K factor, and log mean, by using the proposed method. The true positive rate indicates that the data fell in the LOS condition and the testing results are also in the LOS condition; the false positive rate indicates that the testing results were positive but the data were not. In Figure 7, the results are shown by the data sensitivity; that is, the true positive rate versus the false positive rate. According to the results, the various features were derived with different correction rates for the LOS and NLOS judgments. The worst performing feature was the mean, followed by the log mean, Rician K factor, kurtosis, and our proposed method. In statistics, although the mean value is a useful tool, it eliminates crucial data features. Furthermore, the computations of Rician K factor and kurtosis are based on the appearance of the data, the traits of which are easy to capture. Furthermore, our proposed method is focused on the features; when one considers the time that is consumed, the proposed method outperforms the other approaches on that single feature.

**Figure 8:**
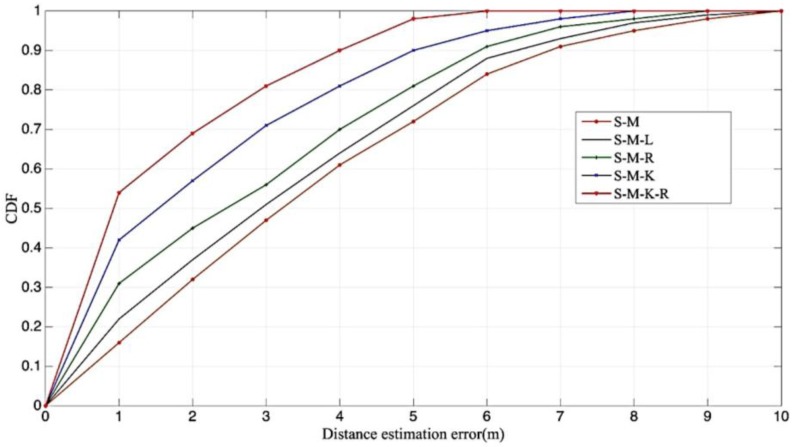
The comparisons of remarkable sets on the features.

Furthermore, to compare the combinations of features, we performed experiments with different joint sets of features. Table 1 defines the joint sets of features. In this experiment, we omitted some unnoteworthy sets, and the comparison of remarkable sets is illustrated in Figure 8. Below 1 m, the estimated distance efficiency rates are accumulated from high to low; the joint feature sets are S-M-K-R, S-M-K, S-M-R, S-M-L, and S-M. The more features a calculation represents correctly, the more accurate that calculation is. The combination features with the mean, kurtosis, and Rician K achieved over 70% efficiency for LOS and NLOS recognition under 1 m.

### 4.3. The Comparisons on the Distinct Situations

**Figure 9:**
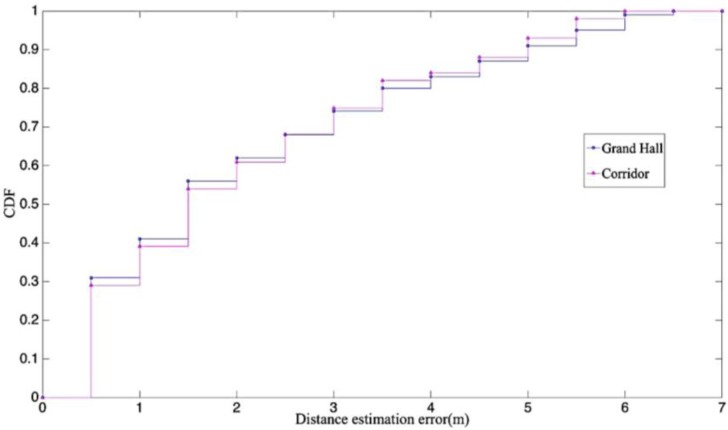
The field experiments on the two environments.

Our field experiments were conducted in two environments, one was a 30 m × 30 m grand hall, the other consisted of corridors with paths that were 40 and 30 m in length. The LOS and NLOS identification results are shown in Figure 9. According to the results, the distance estimation error of grand hall inside 3 m is slightly more accurate than that in the corridor, because the signal in the grand hall had less inflictions on the transmission in the open area. Furthermore, after 3 m, the reflections of signals were more unstable than the signal in the straight pathways, thus the accuracy level was lower in the grand hall.

**Figure 10:**
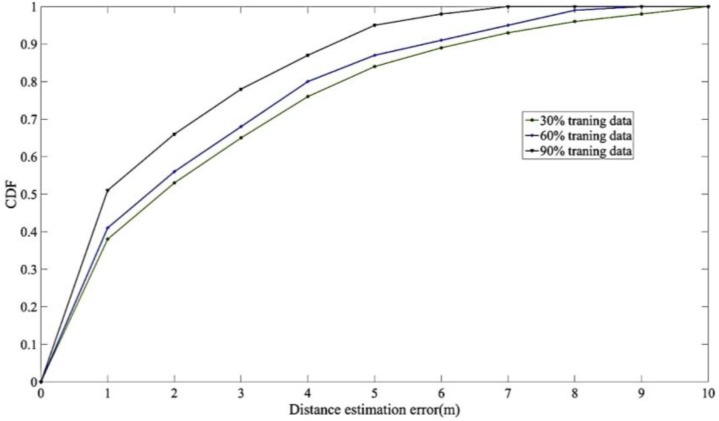
The field experiments on the different size of training samples.

Furthermore, we tested our system on training samples of different sizes. Figure 10 shows that the system was trained on training sets that contained 30%, 60%, and 90% of the total data, and was tested on sets that contained the remaining 70%, 40%, and 10% of the data, respectively. According to the results, the average accuracy errors of the 30%, 60%, and 90% runs were 2.53, 2.47, and 2.31, respectively, and the distance estimation errors insides 1 m were 0.52, 0.54 and 0.66, respectively. The results revealed that the training sample size did not have major effects on the ability of the proposed system to identify information. However, according to the error rate under 1 m, the system with around half training data still obtain the accuracy over than 50%. Hence, the following experiment was conducted on a small training sample to conserve computational effort.

### 4.4. The Robust Testing

**Figure 11:**
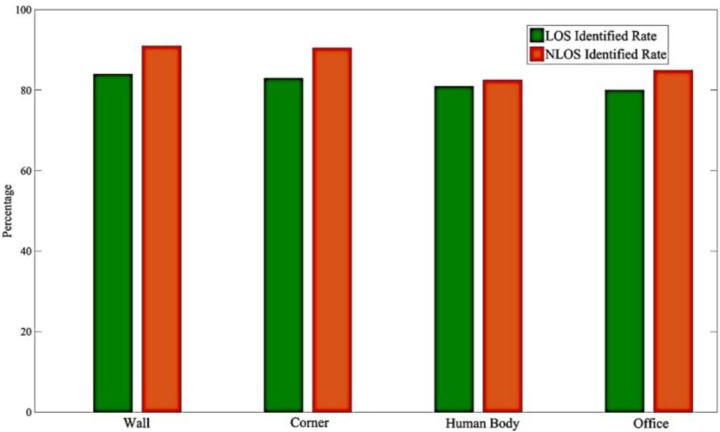
The robust testing on different defilades.

To check the robustness of the proposed method, an experiment was conducted to evaluate the signal blocking. The defilades in our second environment were walls, corners, human bodies, and iron bookshelves. To identify the LOS and NLOS signals, the experiments were performed behind the defilades, and the LOS and NLOS data were collected as in previous experiments. The final results are listed in Figure 11. According to our field tests, the degree of signal blockage does not greatly impair the accuracy of our system. The maximum and minimum average accuracies were respectively 84.3% and 80.41% for the walls and iron bookshelves. Furthermore, concrete defilades, such as walls and corners, produced obvious differences in LOS and NLOS signals.

### 4.5. The Comparisons on the Distinct Models

In this experiment, the accuracy levels of various machine learning methods were considered. Distance estimations for LOS and NLOS identification were conducted with Gaussian process regression (GPR), hypothesis testing regression (HTR), the least squares support vector machine (LS-SVM) methods, and the dynamic RSS fingerprint method proposed in [2], which are described as follows:

LS-SVM is a supervised machine learning algorithm that can be used as a classifier to separate data sets with different features and aspects. To avoid the quadratic programming problem, in [29], LS-SVM was used to simplify the optimization of the weights and misclassification penalties.

HTR can be used to control the equation parameter. LOS and NLOS conditions can vary greatly and can produce significant errors in distance estimation. Therefore, Xiao et al. [6] used HTR to estimate transmitter–receiver distances with different propagation models for LOS and NLOS conditions.

GPR can make probabilistic predictions and infer accurate model hyper-parameters, which can provide precise trade-offs between data fitting and smoothing. Furthermore, low computational complexity makes Gaussian processes suitable for mobile devices with small data sets [30]. According to Xiao et al., GPR is almost identical to the classification with a single difference in the training and testing output. The output in the classification is the LOS and NLOS labels, whereas the regression is used to derive the transmitter–receiver distance.

Dynamic RSS method is the dynamic sampling method proposed by Wen et al. in 2016 [2]. A typical RSS location fingerprint was obtained mainly from a static database, which was ineffective in a dynamic and changeable environment. However, in their study, they improved the traditional RSS fingerprint method by using surrounding feedback information and referring different, newly updated, temporal and spatial RSS of the locations.

**Figure 12:**
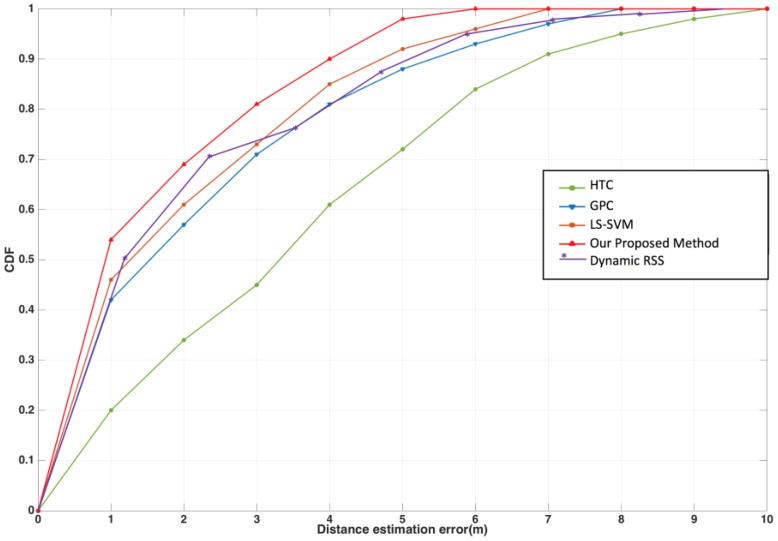
The comparisons with different Machine Learning methods.

Hence, as shown in Figure 12, we derived the LOS and NLOS identifications with LS-SVM, GPR, HTR, the proposed method and the dynamic RSS method with average estimation errors of 3.85, 2.86, 2.77, 2.21 and 2.45 m, respectively. According to this result, HTR obtained an unfavorable prediction result, mainly because that method is based on linear classification, but the nonlinear LOS and NLOS signals are extremely complex. Furthermore, LS-SVM and GPR can choose the nonlinear kernels to improve prediction accuracy, but the trade-off is that they extend the computation time considerably. Besides, comparing to the dynamic RSS method proposed by the author in 2016 [2], although the computation process in this study is complicated than the dynamic RSS method, however, the distinct features can bring the flexibility on the interval comparisons, the more considerations we got, the more chance to increase the accuracy of prediction. Thus, observing to the line trend in Figure 12, inside 1 m, our proposed method has more chance to predicate correctly, and the total accuracy still surpassed the dynamic RSS method. Generally, the proposed method adopts different features with different weights, and computes them with flexible interval computation; the feature symbolism can be retained by measuring the strong evidence.

## 5. Conclusions

In this paper, we propose new methods on the basis of belief intervals, as proposed in DS theory, to address the problem of LOS and NLOS identification with RSS measurement. DS theory is well-known for its usefulness in expressing the uncertain judgments of experts. To our knowledge, this is the first belief interval application for LOS and NLOS identification in an indoor positioning system. It is difficult to derive exact numbers in LOS and NLOS identification, especially because various irrelevant signals can interfere with the signals in LOS and NLOS situations, and because defilades also block signals in the environment. Thus, to reduce the negative influence of signal side effects, a novel approach to DS theory’s rule of combination is proposed to support the classification of key features from various collected RSS measurements useful for identifying LOS/NLOS condition, including the mean, standard deviation, kurtosis, skewness, and Rician K factor. All features are derived from the RSS sampled from the beacons at a particular location over a short period.

Because our system is based on offline training, the main limitation of the proposed method is the cost of the training phase. To reduce the complexity of system initialization, the maps and beacon locations should be considered in advance. Furthermore, computation of online comparisons can be eliminated by using integrated equations. According to our experiments, signal features produce different performance levels, and the proposed system outperformed the Machine Learning algorithm, like GPR, HTR, LS-SVM and Dynamic RSS methods. The advantage of the proposed system is the weighted derivation of a DS belief interval, which can reduce the computational complexity and identify the features of LOS and NLOS signals. Although the fingerprinting localization system had limited performance in the past, and our proposed method has suffered the difficulties on the construction of the offline database and the environment/AP/Beacons changed. However, the new feature extraction framework of our proposed method, which can identify the original signals in the obstacle environment and improve the localization precision stability.

## Figures and Tables

**Table 1:** Remarkable sets of features.

Joint Sets	Mean (M)	Kurtosis (K)	Rician K Factor (R)	Log-Mean (L)
**S-M**	√			
**S-M-L**	√			√
**S-M-R**	√		√	
**S-M-K**	√	√		
**S-M-K-R**	√	√	√	
